# Pathway and Production Differences in Branched-Chain Hydroxy Acids as Bioactive Metabolites in *Limosilactobacillus fermentum*, *Ligilactobacillus salivarius*, and *Latilactobacillus sakei*

**DOI:** 10.3390/ijms251810112

**Published:** 2024-09-20

**Authors:** Dong-Hyuk Kim, Su-Hyun Kim, Seul-Ah Kim, Min Jeong Kwak, Nam Soo Han, Choong Hwan Lee

**Affiliations:** 1 Department of Bioscience and Biotechnology, Konkuk University, Seoul 05029, Republic of Korea; 2 Brain Korea 21 Center for Bio-Health Industry, Division of Animal, Horticultural, and Food Sciences, Chungbuk National University, Cheongju 28644, Republic of Korea

**Keywords:** Lactobacillaceae, bioactive metabolites, probiotics, metabolomics, 2-hydroxy isovaleric acid (HIVA), 2-hydroxy isocaproic acid (HICA), 2-hydroxy-3-methyl valeric acid (HMVA)

## Abstract

Branched-chain hydroxy acids (BCHAs) as bioactive metabolites of Lactobacillaceae include 2-hydroxy isovaleric acid (HIVA), 2-hydroxy isocaproic acid (HICA), and 2-hydroxy-3-methyl isovaleric acid (HMVA). Combining targeted and untargeted metabolomics, this study elucidates differences in extracellular BCHA production in *Limosilactobacillus fermentum*, *Ligilactobacillus salivarius*, and *Latilactobacillus sakei* alongside comparing comprehensive metabolic changes. Through targeted metabolomics, BCHA production among 38 strains exhibited strain specificity, except for *L. sakei*, which showed significantly lower BCHA production. Explaining the lower production in *L. sakei*, which lacks the branched-chain amino acid (BCAA)-utilizing pathway, comparison of BCHA production by precursor reaction revealed that the pathway utilizing BCAAs is more dominant than the pathway utilizing pyruvate. Expanding upon the targeted approach, untargeted metabolomics revealed the effects of the reaction compound on other metabolic pathways besides BCHAs. Metabolism alterations induced by BCAA reactions varied among species. Significant differences were observed in glycine, serine, and threonine metabolism, pyruvate metabolism, butanoate metabolism, and galactose metabolism (*p* < 0.05). These results emphasize the importance of the synergy between fermentation strains and substrates in influencing nutritional components of fermented foods. By uncovering novel aspects of BCAA metabolism pathways, this study could inform the selection of fermentation strains and support the targeted production of BCHAs.

## 1. Introduction

Lactobacillaceae, a prominent family of probiotic microorganisms, is utilized in food fermentation and resides in the intestinal environment. It produces various bioactive metabolites, showing positive effects on pathogenic bacteria control, weight control, and gut barrier improvement [1,2]. Among these bioactive metabolites, branched-chain hydroxy acids (BCHAs), specifically 2-hydroxy isovaleric acid (HIVA), 2-hydroxy isocaproic acid (HICA), and 2-hydroxy-3-methyl isovaleric acid (HMVA), have recently garnered attention. Our previous research confirmed that HIVA and HICA promote the growth of beneficial gut bacteria while inhibiting the growth of pathogenic bacteria [3]. Research on the physiological effects of BCHAs has demonstrated their ability to enhance insulin action on glucose metabolism in liver and muscle cells [4]. Furthermore, studies have explored antifungal properties of HIVA and its influence on the proliferation of intestinal epithelial cells [5,6]. Additionally, research has been conducted on antibacterial and antifungal effects of HICA [7,8]. A remarkable aspect is that the health-beneficial efficacy of BCHAs has been documented in separate studies, and the accumulated research in recent years has drawn attention to the classification of BCHAs, which share a production pathway. The biological pathway for BCHA synthesis in Lactobacillaceae has been elucidated through previous studies [9]. In this pathway, the enzymes BcaT and AraT play a role in converting BCAAs (valine, leucine, and isoleucine) into keto acid intermediates (2-ketoisovaleric acid, 2-ketoisocaproic acid, and 2-keto-3-methylvaleric acid), with BcaT being identified as a significant contributor [10]. Generated keto acids undergo conversion into branched-chain hydroxy acids through the action of the Hycdh enzyme, consequently leading to the synthesis of BCHAs. Another BCHA synthesis pathway utilizing pyruvate as a precursor follows a series of reactions shared with the BCAA synthetic pathway, culminating in the production of keto acids and subsequently resulting in the formation of BCHAs [11]. However, the presence or absence of enzyme-expressing genes involved in the pathway varies by species; in particular, *L. sakei* lacks the BcaT- and AraT-coding genes [12,13]. While these variances might affect BCHA production, no studies have yet been conducted to address and compare this aspect. Moreover, given the continuous emergence of research findings suggesting the diverse health benefits of BCHAs, there is a requirement for insights about production and pathway.

Targeted metabolomics is the optimized method for comparing the production of particular metabolites and those encompassed within the pathways related to them. Targeted metabolomics optimizes the metabolite extraction and analysis methods for the accurate detection of particular compounds of interest. In contrast, untargeted metabolomics is an unbiased approach to comprehensively profile low-molecular-weight metabolites (<1800 Da) including all compounds in a biological system. Untargeted metabolomics has the potential to offer valuable insights into metabolism by enabling the detection of broad-ranging metabolic alterations. This combination of two strategies enables precise evaluations of target compounds, while also allowing the exploration of the whole metabolite profile [14,15]. The present study applied this combination to a cultivation experiment and reaction mixture experimental model. Reaction mixture experiments were utilized in studies investigating specific substrate-related pathways. This experiment involves culturing cells initially cultured in complex media after washing, then cultivating them in pure cultures with defined substrates [16,17].

We aspired to elucidate BCHA production differences in cultivation experiments, to further validate pathway differences through reaction mixture experiments. Experiments were performed using selected strains of Lactobacillaceae, including *Limosilactobacillus fermentum*, *Ligilactobacillus salivarius*, and *Latilactobacillus sakei*, known for their variations in BCHA pathway enzyme-encoding genes. Targeted metabolomics through gas chromatography–time of flight–mass spectrometry (GC-TOF-MS) was utilized to compare BCHA production of these Lactobacillaceae species. Furthermore, untargeted metabolomics was performed to analyze metabolic changes under BCAA and pyruvate reaction conditions. To interpret a wide range of metabolic alterations, untargeted metabolomics was conducted using GC-TOF-MS and ultra-high-performance liquid chromatography–Orbitrap–tandem mass spectrometry (UHPLC–Orbitrap–MS/MS) instruments, which offer complementary detection of compounds.

## 2. Results and Discussion

### 2.1. Strain-Specific Variations in BCHA Production among 38 Lactobacillaceae Strains Are Unveiled through Targeted Metabolomics

**Figure 1:**
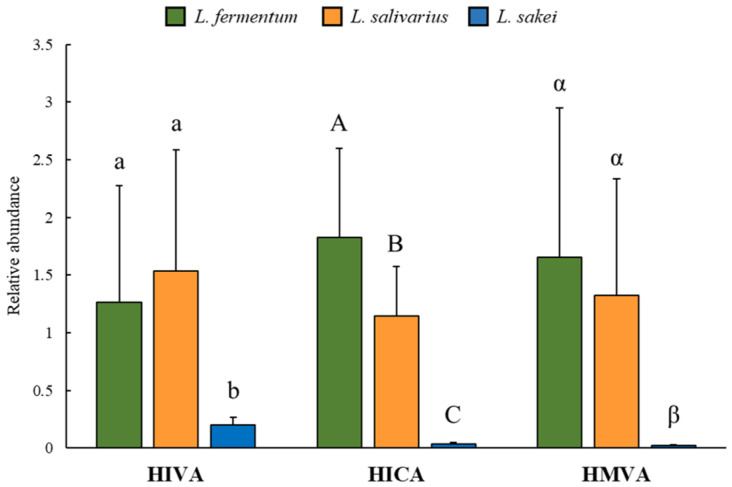
Branched-chain hydroxy acid (BCHA) production in MRS cultivation. Different letters in the bar graph indicate a significant difference determined by ANOVA followed by Duncan’s multiple-range test (*p* < 0.05).

To evaluate BCHA production under cultivation conditions, we conducted targeted metabolomics of BCHAs in an extracellular environment of 38 strains cultivated under MRS media. A noteworthy observation was that *L. sakei* displayed a statistically low production level (Figure 1). In the strain data, all strains of *L. sakei* exhibited significantly lower HIVA production than the other species. *L. fermentum* and *L. salivarius* showed significant strain-level variations even within the same species (Supplementary Figure S1). Strain specificity was also evident in HICA and HMVA production, with *L. sakei* showing a significantly low production level. The deficiency of BcaT- and AraT-coding genes in *L. sakei* is responsible. This was confirmed at the metabolic level using precursor reaction experiments detailed in Section 2.2.

Both *L. fermentum* and *L. salivarius* contain the BcaT- and AraT-coding genes. Prior research on lactic acid bacteria has highlighted substrate specificity variations in aminotransferase enzymes, including BcaT and AraT, across bacterial strains [18,19,20]. Based on this, we expect that the production of BCHA by lactic acid bacteria will also exhibit strain specificity. Despite the strain-specific nature of BCHA production, we observed a distinctive clustered pattern between species in extracellular metabolomes using PCA (Supplementary Figure S2). These results exemplify the strain-specific nature of selecting probiotics [21]. Although the metabolomes of the species are similar, the variation in bioactive metabolite production underscores the importance of strain-level selection. By comparing the BCHA production levels of different strains, these findings offer potential applications for food fermentation and industrial purposes.

### 2.2. Species-Specific Variations in BCHA Pathway Were Validated in Reaction Mixture through Targeted Metabolomics

The biological pathway for BCHA synthesis in Lactobacillaceae has been elucidated in prior studies [9]. In this experiment, we validated the differences in this pathway through targeted metabolomics based on the related genetic studies. Additionally, we identified the metabolic pathways that exert a greater influence on BCHA production through a comparison of pathways corresponding to their precursors. We compared three strains in an extracellular environment incubated under reaction-mixture-supplemented precursors, such as BCAAs and pyruvate.

According to previous genomics studies, *L. fermentum* and *L. salivarius* both express BcaT and AraT genes, while *L. sakei* subsp. *sakei* lacks both genes [12,13]. We utilized one strain per species, namely *L. fermentum* EFEL 6804 (LFE 6804), *L. salivarius* KGMB 2057 (LSL 2057), and *L. sakei* subsp. *sakei* KACC 12414 (LSK 12414), from the previous cultivation experiment. Based on genetic studies, we suggested that while all strains would convert pyruvate into BCHAs, LFE 6804 and LSL 2057 would be capable of converting BCAAs into BCHAs, whereas LSK 12414 would not exhibit this capability. As anticipated, all strains converted pyruvate to BCHAs, and LFE 6804 and LSL 2057 produced BCHAs from the BCAA precursor. In contrast, LSK 12414, lacking BcaT- and AraT-coding genes, did not produce BCHAs during the BCAA reaction.

**Figure 2:**
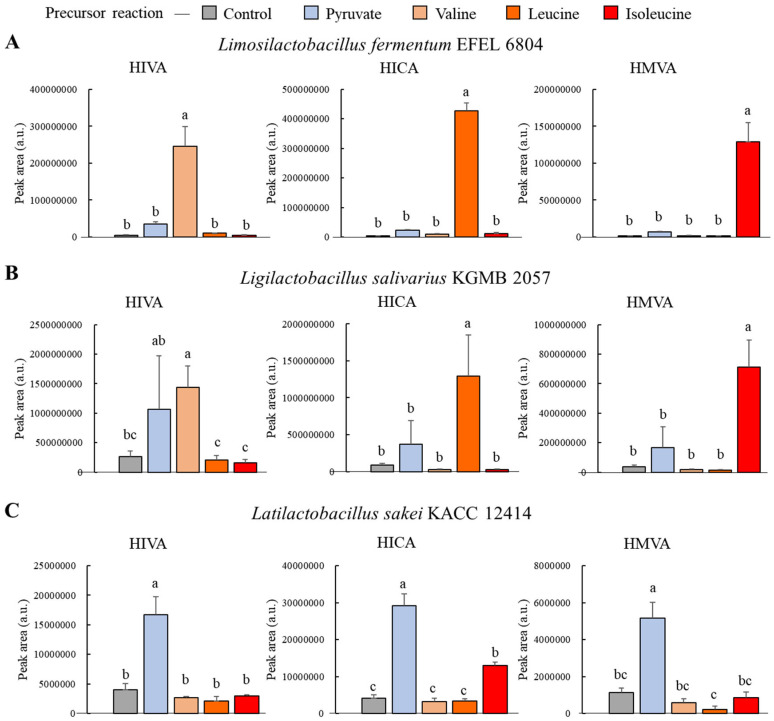
Branched-chain hydroxy acid (BCHA) production in the reaction mixture experiments. (**A**) *Limosilactobacillus fermentum* EFEL 6804, (**B**) *Ligilactobacillus salivarius* KGMB 2057, and (**C**) *Latilactobacillus sakei* KACC 12414. Different letters in the bar graph indicate a significant difference determined by ANOVA followed by Duncan’s multiple-range test (*p* < 0.05).

LFE 6804 exhibited statistically significant increases in BCHA production corresponding to the BCAA precursor reaction compared to the non-reaction control group. Specifically, HIVA showed a 46.98-fold increase, HICA exhibited a 102.02-fold increase, and HMVA demonstrated an 86.95-fold increase (Figure 2A). The pyruvate reaction, when compared to the non-reaction control group, showed statistically insignificant increases, with HIVA increasing 6.69-fold, HICA 5.25-fold, and HMVA 5.25-fold.

LSL 2057 exhibited statistically significant increases in BCHA production corresponding to the BCAA precursor reaction compared to the non-reaction control group. Specifically, HIVA showed a 5.38-fold increase, HICA exhibited a 14.68-fold increase, and HMVA demonstrated an 18.83-fold increase (Figure 2B). The pyruvate reaction, when compared to the non-reaction control group, showed statistically insignificant increases, with HIVA increasing 3.98-fold, HICA 4.23-fold, and HMVA 4.44-fold.

LSK 12414, which lacked the BcaT and AraT enzyme coding genes, did not increase BCHA production corresponding to the BCAA precursor reaction (Figure 2C). The pyruvate reaction, when compared to the non-reaction control group, showed statistically significant increases, with HIVA increasing 4.15-fold, HICA 7.12-fold, and HMVA 4.44-fold.

**Figure 3:**
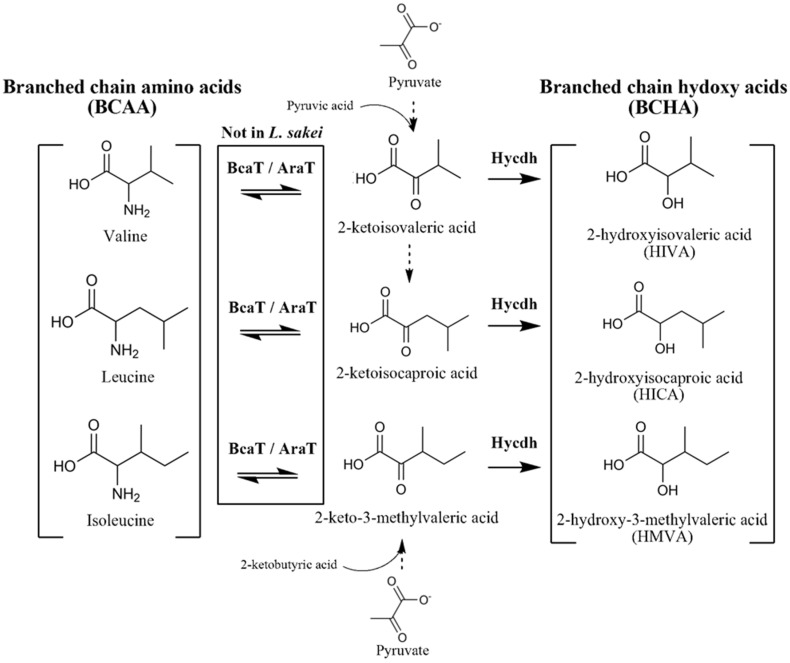
Pathway difference in the branched-chain hydroxy acids (BCHAs) in *Limosilactobacillus fermentum*, *Ligilactobacillus salivarius*, and *Latilactobacillus sakei*.

The increase in BCHAs resulting from the pyruvate reaction showed an approximately five-fold increase in all strains and products. In contrast, the increase in BCHAs resulting from the BCAA reaction in LFE 6804 was approximately 50 to 100 times, in LSL 2057 approximately 5 to 20 times, and in LSK 12414, it did not increase as expected. These results validated differences in the Lactobacillaceae BCHA pathway based on the presence or absence of these genetic elements (Figure 3). Additionally, it was confirmed that the pathway containing BcaT and AraT plays a major role in BCHA production, utilizing BCAAs as a precursor, and also contributes the difference in BCHA production. Although the significant role of BCHAs in probiotic efficacy has been sequentially revealed, previous studies on the BCHA metabolic pathway did not try to demonstrate inter-species differences at the metabolism level [18,19,20]. The present study addressed this gap by utilizing a combination of targeted metabolomics and genomics references.

### 2.3. Metabolomic Differences in Pyruvate and BCAA Reaction Mixtures of L. fermentum EFEL 6804, L. salivarius KGMB 2057, and L. sakei KACC 12414 Were Explored through Untargeted Metabolomics

We previously explored variations in BCHA production and pathway of *L*. *fermentum*, *L. salivarius*, and *L. sakei*. Expanding on this, one strain per species was selected to analyze metabolomic differences in the untargeted metabolomics of BCHA precursor reaction mixtures. Pyruvate and BCAAs can alter various metabolic pathways beyond BCHA production. We anticipated that these changes would vary among different species. The extracellular metabolic changes induced by each compound will provide valuable insights into the impact on the nutritional components of substrates in food fermentation. To comprehensively interpret metabolomic changes, we employed two complementary instruments, GC-TOF-MS and UHPLC–Orbitrap–MS/MS. Multivariate statistical analysis facilitated extensive metabolomic comparisons, while heatmap and pathway analysis allowed for a detailed examination of metabolic alterations.

#### 2.3.1. Metabolite Profiles in Pyruvate and BCAA Reaction Mixtures of *L. fermentum* EFEL 6804, *L. salivarius* KGMB 2057, and *L. sakei* KACC 12414 Were Visualized by Principal Component Analysis

To visualize differences in metabolism in pyruvate and BCAA reaction mixture among these three Lactobacillaceae species, principal component analysis (PCA) was carried out. An initial exploratory PCA of the dataset revealed that for GC-TOF-MS (Supplementary Figure S3A), the first and second components (PC1 and PC2) accounted for 6.74% and 5.69% of the variance, respectively. The PCA graph indicated separation of three strains on the graph by two principal components. An intriguing observation was that BCAA reaction groups formed clusters in all strains, suggesting similar metabolism within BCAA reaction groups of each strain. PCA results for the UHPLC–Orbitrap–MS/MS positive-ion-mode dataset showed that PC1 and PC2 explained 14.9% and 11.6% of the variance, respectively (Supplementary Figure S3B). In PCA analysis of the UHPLC–Orbitrap–MS/MS negative-ion-mode dataset, PC1 and PC2 accounted for 14.6% and 9.19% of the variance, respectively (Supplementary Figure S3C). Similar to the GC-TOF-MS dataset, both ion mode datasets of UHPLC–Orbitrap–MS/MS exhibited clustering among BCAA reaction groups for each strain.

These consistent outcomes diverged from findings of previously described BCHA target analysis. Specifically, LFE 6804 and LSL 2057 exhibited varying BCHA production for each BCAA reaction, yet overall metabolism remained similar among BCAA reaction groups. Based on results of unsupervised multivariate statistical analysis, we utilized a heatmap to represent and comparatively analyze metabolic distinctions among reaction mixtures specific to each microbial strain.

#### 2.3.2. Metabolic Differences in Pyruvate and BCAA Reaction Mixtures of *L. fermentum* EFEL 6804, *L. salivarius* KGMB 2057, and *L. sakei* KACC 12414 Were Visualized by a Heatmap and a Pathway Map

**Figure 4:**
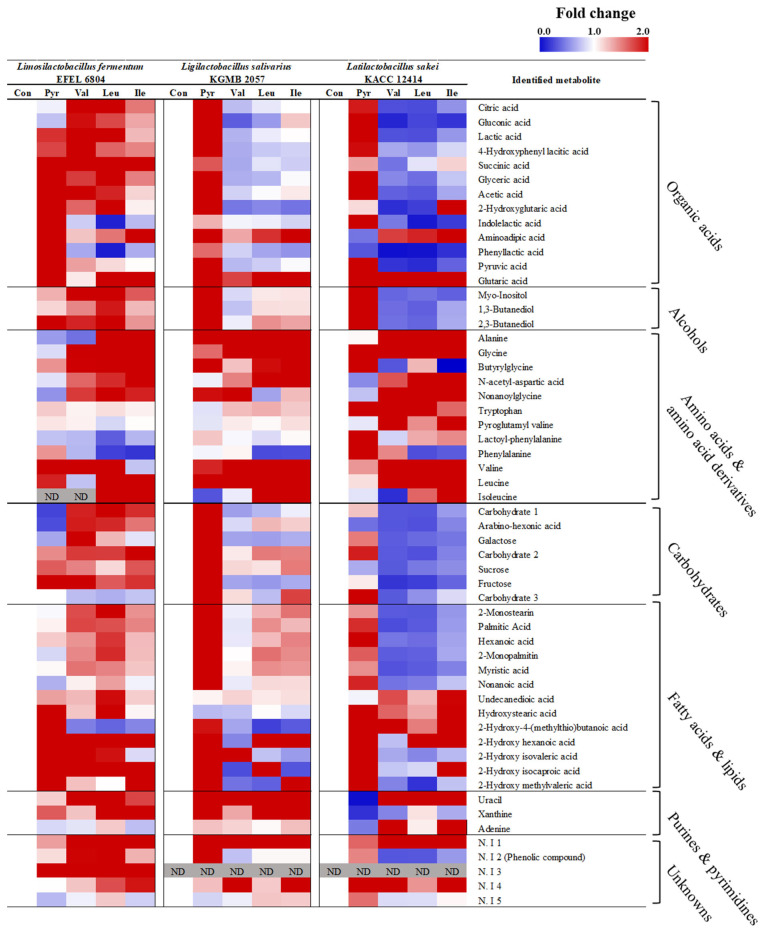
Untargeted metabolomics heatmap of the reaction mixture experiment. The relative metabolite levels were normalized to non-reaction control groups specific to each strain. All metabolites were significantly distinguished in each group by one-way analysis of variance (*p* < 0.05).

**Figure 5:**
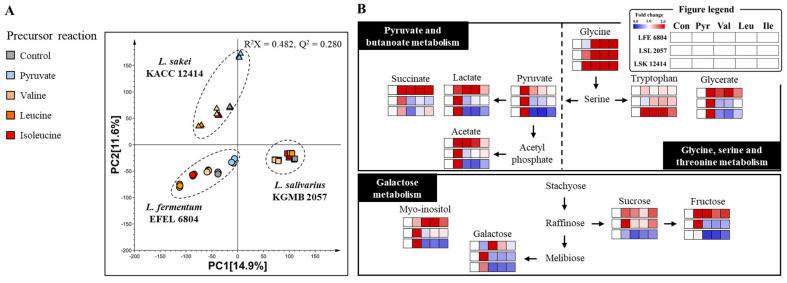
(**A**) Principal component analysis of the reaction mixture experiment analyzed by UHPLC–Orbitrap–MS/MS in positive ion mode. (**B**) Significantly altered metabolism pathways under BCHA precursor reactions. LFE 6804, *Limosilactobacillus fermentum* EFEL 6804; LSL 2057, *Ligilactobacillus salivarius* KGMB 2057; LSK 12414, *Latilactobacillus sakei* KACC 12414.

Metabolite identification was conducted to discern significant differences among reaction mixtures within each microbial strain (Supplementary Tables S1 and S2). Visualization of these metabolites, normalized to non-reaction control groups specific to each strain, was achieved through a heatmap (Figure 4). As observed in Supplementary Figure S2 and Figure 5A, metabolic changes across the three strains exhibited similarity within the BCAA reaction group. Organic acids such as acetate, lactate, glycerate, citrate, pyruvate, succinate, and gluconate increased in the pyruvate reaction samples of all strains, as well as in the BCAA reaction samples of LFE 6804, whereas they decreased in the BCAA reaction sample of LSK 12414. Carbohydrates such as galactose, sucrose, fructose, and myo-inositol increased in the pyruvate reaction sample of LSL 2057 and the BCAA reaction sample of LFE 6804, while they decreased in the BCAA reaction sample of LSK 12414. Aromatic amino acids and derivatives such as tryptophan, phenylalanine, indolelactic acid, phenyllactic acid, and 4-hydroxyphenyllactic acid increased in the pyruvate reaction sample of LFE 6804. Fatty acids and lipids such as hexanoic acid, myristic acid, nonanoic acid, palmitic acid, monosteain, and monopalmitin increased in the pyruvate reaction samples of LSL 2057 and LSK 12414 and the BCAA reaction sample of LFE 6084, whereas they decreased in the BCAA reaction sample of LSK 12414. Valine, leucine, isoleucine, HIVA, HICA, and HMVA, components of branched-chain amino acid metabolism, showed an increase in the supplemented BCAAs in each sample and an alteration in product formation across different strains according to the BCHA pathway.

Leveraging insights from our prior investigations [22,23], which revealed variability in Lactobacillaceae metabolism based on cultivation conditions and strains, we applied these observations to unravel metabolism alteration specific to reaction compounds in the current study. Pathway enrichment analysis was conducted to determine the metabolic pathways significantly affected by the reaction compounds (Supplementary Figure S4). The alterations in metabolite levels within these significant pathways were visualized on a pathway map (Figure 5B) and summarized in Table 1. A shared pattern observed across the three Lactobacillaceae strains was overall stimulation of metabolism in the pyruvate reaction mixture. Pyruvate, a compound node generated through the EMP pathway, functions as a carbon source that is directly converted into various compounds. Additionally, it influences gene expression and enzyme activity, thereby regulating metabolism [24,25]. Metabolic acceleration triggered by pyruvate in our study aligned with earlier findings such as induction of fatty-acid-biosynthesis-related genes (fabH, accCm, and fabI) in *L. bulgaricus* due to pyruvate metabolism and increased expression of genes and proteome activity related to the EMP pathway, sugar metabolism, and amino acid synthesis in *L. helveticus* under elevated carbon source concentrations [26,27].

An intriguing observation emerged in the BCAA reaction mixtures of these strains. In the metabolism of glycine, serine, and tryptophan, an increase in glycine and tryptophan was observed across all strains. Additionally, glycerate levels significantly increased only in strain LFE 6804. Lactate and acetate, within the pathways of butanoate and pyruvate metabolism, showed a significant increase in the LFE 6804 strain, whereas a decrease was observed in the LSK 12414 strain. In this study, the observed variations in metabolite levels between strains are likely attributed to transcriptional regulation and enzyme activity influenced by BCAAs. Supporting studies indicate that Lactobacillaceae undergoes transcriptional and enzyme activity changes across diverse metabolisms in response to BCAAs. Based on a comprehensive review of previous studies, these influences are expected to exhibit a tendency to be strain-specific rather than species-specific. The observed differences are attributable to disparities in amino acid transport and the expression levels of certain metabolic enzymes. Strain-specific differences in amino acid transport have been identified in *L. helveticus* [28]. Studies have shown that the regulation of amino acid transporters and metabolic enzymes in *L. amylovorus* is affected under culture conditions supplemented with free amino acids, including valine and leucine [29]. Jing and colleagues also demonstrated that the expression of ackA, an enzyme involved in acetate production, and ldh, an enzyme involved in lactate production in *L. amylovorus*, is regulated by the supplementation of free amino acids in the environment. Additionally, amino acid supplementation studies with *L. casei* have demonstrated regulation of the EMP pathway and tricarboxylic acid cycle at both metabolite and transcription levels [30]. The regulation of proteolytic system gene expression by BCAA binding with transcriptional regulators is reported in *L. helveticus* [31]. These studies provide a basis for explaining the strain-specific utilization and metabolic alterations observed in the BCAA reactions of this study. The consistency of our results with prior research, conducted at transcription and enzyme levels, underscores the significance of the metabolomic approach. These findings support the hypothesis that the bacterial capability of resource or nutrient utilization could synergize between fermentation strains and substrates in influencing nutritional components of fermented foods.

The reactions of pyruvate and BCAA regulated metabolic pathways beyond BCHA production, with strain-specific impacts in glycine, serine, and threonine metabolism, pyruvate metabolism, butanoate metabolism, and galactose metabolism. In this manner, there are metabolic differences among Lactobacillaceae based on growth environment, species, and strain, yet there are inherent limitations in comprehending these variances [21,32,33]. As previously mentioned, transcriptional regulation is also a major contributor to metabolic alterations, emphasizing the importance of synergy between metabolism and transcriptomics [34]. To the best of our knowledge, there has been no research comparing the transcriptome of the strain used in this study or members of Lactobacillaceae under BCAA-supplemented conditions. Future transcriptome studies are anticipated to interpret these results from an integrated omics perspective. Nevertheless, as evidenced by this research, conducting both targeted and untargeted approaches concurrently can enrich the depth of conclusions and facilitate a comprehensive understanding. In subsequent research endeavors, utilizing both targeted and untargeted approaches could broaden the insights of probiotics metabolism. This expanded understanding could serve as crucial knowledge for contributing to human health regarding probiotics and bioactive metabolites.

## 3. Materials and Methods

### 3.1. Chemicals and Reagents

HPLC-grade water, methanol, and acetonitrile were procured from Thermo Fisher Scientific (Waltham, MA, USA). Sodium pyruvate, L-valine, L-leucine, L-isoleucine, N-methyl-N-(trimethylsilyl) trifluoroacetamide (MSTFA), methoxyamine hydrochloride, pyridine, and phosphate-buffered saline (PBS) were purchased from Sigma-Aldrich (St. Louis, MO, USA). DeMan, Rogosa, and Sharpe (MRS) broths were obtained from DIFCO Laboratories Inc. (BD Biosciences, Franklin Lakes, NJ, USA).

### 3.2. Bacterial Strains and Culture Conditions

A total of 38 strains of Lactobacillaceae were used in this study (Table 2). These strains were procured from several sources, including the Korean Culture Center of Microorganisms (KCCM, Seoul, Republic of Korea), the Korean Collection for Type Cultures (KCTC, Jeongeup, Republic of Korea), the Korean Gut Microbiome Bank (KGMB, Jeongeup, Republic of Korea), the Korean Agricultural Culture Collection (KACC, Wanju, Republic of Korea), and generously provided by Professor Nam Soo Han from the Department of Food Science and Technology at Chungbuk National University (Cheongju, Republic of Korea).

#### 3.2.1. MRS Cultivation Experiment

All strains were inoculated in 14 mL of an MRS medium supplemented with 0.05% L-cysteine in a 15 mL conical tube with a 0.14 mL bacterial suspension (optical density [OD] at 600 nm, 1.0) at 37 °C for 24 h in an anaerobic chamber (Coy Laboratory Products, Grass Lake, MI, USA) containing 10% CO_2_, 5% H_2_, and 85% N_2_. Subsequently, cultures were immediately centrifuged at 11,011 g for 10 min at 4 °C and filtered through a 0.2 μm polytetrafluoroethylene (PTFE) filter (Hyundai micro, Ansung, Republic of Korea) to disunite cell-free supernatant (extracellular metabolome) for extraction.

#### 3.2.2. Reaction Mixture Experiment

Table 2 lists three strains marked with asterisks utilized in the reaction mixture experiment. Forty milliliters of MRS medium supplemented with 0.05% L-cysteine in a 50 mL conical tube (Eppendorf, Hamburger, Germany) were inoculated with a 0.4 mL bacterial suspension (optical density [OD] at 600 nm: 1.0) and then incubated at 37 °C for 24 h in an anaerobic chamber. Subsequently, cultures were immediately centrifuged at 11,011 g for 10 min at 4 °C to harvest cells. Cell pellets were washed twice with pH 5.5 PBS (1X) for precursor reactions. Ten milliliters of pH 5.5 PBS (1X) containing 7 mM each of pyruvate, valine, leucine, and isoleucine were added to washed cell pellets. As a control sample, pH 5.5 PBS (1X) without precursors was used. After incubation at 37 °C for 24 h in an anaerobic chamber, cultures were immediately centrifuged at 11,011 g for 10 min at 4 °C and filtered through a 0.2 μm polytetrafluoroethylene (PTFE) filter to disunite cell-free supernatant (extracellular metabolome) for extraction.

### 3.3. Extraction of Metabolites

Five milliliters of separated supernatant were aliquoted and extracted with 10 milliliters of 50% MeOH in water solvent containing 10 μg/mL of internal standard (2-chlorophenylalanine) at 25 °C for 24 h. Supernatants were filtered through a 0.2 μm polytetrafluoroethylene (PTFE) filter and dried using a speed vacuum concentrator, Modulspin 31 (Biotron, Wonju, Republic of Korea). To ensure the stability of the dried samples, they were kept in the dark at deep-freezing conditions (−20 °C) and subjected to MS analysis within one week.

### 3.4. GC-TOF-MS Analysis

Dried samples were reconstituted with derivatization reagent (5 mg/mL). For the derivatization process, dried samples were oximated with 50 µL of methoxyamine hydrochloride (20 mg/mL in pyridine) for 90 min at 30 °C and silylated with 50 µL of N-methyl-N-(trimethylsilyl) trifluoroacetamide (MSTFA) for 30 min at 37 °C. All samples were filtered using a Millex GP 0.22 μm filter (Merck Millipore, Billerica, MA, USA) prior to instrument analysis. GC-TOF-MS analysis was performed using an Agilent 7890A GC system with an Agilent 7693 autosampler and a Pegasus HT TOF-MS. An Rtx-5MS capillary column (30 m × 0.25 mm × 0.25 μm particle size, Restek Corp., Bellefonte, PA, USA) was used. Operational parameters were adapted from a previous study [35]. A total of 1 μL of derivatized samples was injected into GC-TOF-MS with a split ratio of 10:1. All analyses were performed in a random order to reduce bias and conducted in triplicate.

### 3.5. UHPLC–Orbitrap–MS/MS Analysis

For UHPLC–Orbitrap–MS/MS analysis, dried samples were reconstituted in 50% MeOH in water (5 mg/mL), filtered through a 0.2 μm PTFE filter, and then subjected to analysis. The samples were analyzed using Orbitrap Exploris 120 (Thermo Fischer Scientific, Waltham, CA for USA) equipped with a Vanquish Core HPLC system (Thermo Fisher Scientific) and a Phenomenex KINETEX^®^ C18 column (100 mm × 2.1 mm, 1.7 μm particle size; Phenomenex, Torrance, CA, USA) for chromatographic separation. Mobile phases consisted of 0.1% formic acid in water (A) and acetonitrile (B). Compositions of gradient flows were the same. The gradient was gradually increased from 5% solvent B to 100% solvent B over 9 min. It was then maintained for a further 1 min. The flow rate was 0.3 mL min^−1^. Mass spectra were obtained using electrospray ionization in positive and negative ion mode with tandem mass scan modes within a range of *m*/*z* 100–1500. Operating parameters were as follows: spray voltage, 3.5 kV in positive mode and 2.8 kV in negative mode; ion transfer tube temperature, 320 °C; vaporizer temperature, 300 °C; radio frequency (RF) lens level, 70%; resolution, 60,000. All analyses were performed in a random order to reduce bias and conducted in triplicate.

### 3.6. Data Processing and Statistical Analysis

GC-TOF-MS data were acquired and preprocessed using LECO Chroma TOF™ software (version 4.44, LECO Corp., St. Joseph, MI for USA). They were converted into a NetCDF format (*.cdf) using the LECO Chroma TOF™ software. After conversion, peak detection, retention time correction, and alignment were processed using the Metalign software package “http://www.metalign.nl (accessed on 18 September 2024)”. The heatmap and BCHA production bar graphs were rendered using the relative peak area of aligned unique masses with Metalign. UHPLC–Orbitrap–MS/MS data were acquired and preprocessed using Thermo freestyle software (version 1.8.63.0, Thermo Fisher Scientific) and converted into abf format (*.abf) using ABF converter software “https://www.reifycs.com/abfconverter/ (accessed on 18 September 2024)”. After conversion, peak detection, retention time correction, and alignment were processed using MS dial software (version 5.1.230807). Statistical significance of GC-TOF-MS analysis and UHPLC–Orbitrap–MS/MS analysis results was determined by analysis of variance (ANOVA) and Duncan’s multiple-range tests using PASW Statistics 18 software (SPSS Inc., Chicago, IL, USA). Principal component analysis (PCA) was conducted using SIMCA-P+ version 12.0 (Umetrics, Umeå, Sweden). Pathway enrichment analysis was performed using the MetaboAnalyst 6.0 web-based platform [36]. The input data were auto-scaled and examined against the *Latilactobacillus sakei* subsp. *sakei* 23K KEGG pathway library, using global test methods.

## 4. Conclusions

This study comparatively analyzed production and pathway differences in BCHAs in three Lactobacillaceae species. Combining targeted and untargeted metabolomics, this study elucidates differences in BCHA production in Lactobacillaceae alongside comparing comprehensive metabolic changes. Through targeted metabolomics, we compared the BCHA production among 38 strains of Lactobacillaceae in cultivation media. BCHA production among 38 strains exhibited strain specificity, except for *L. sakei*, which showed significantly lower BCHA production. In an effort to explain this discrepancy, we suggested that the presence or absence of enzyme-expressing genes within the pathway, particularly AraT and BcaT, would be influential. Following this, validation was performed through precursor reaction mixture experiments. *L. fermentum* EFEL 6804 and *L. salivarius* KGMB 2057 converted BCAAs into BCHAs, while the *L. sakei* KACC 12414 strain failed to convert BCAAs into BCHAs. These results enabled the validation of the major pathway for Lactobacillaceae BCHA production and the confirmation of production variation resulting from these differences. Expanding upon the targeted approach, untargeted metabolomics revealed the effects of the reaction compound on other metabolic pathways besides BCHAs. Metabolism alterations induced by BCAA reactions varied significantly among species. Significant differences were observed particularly in glycine, serine, and threonine metabolism, pyruvate metabolism, butanoate metabolism, and galactose metabolism. These findings support the hypothesis that the bacterial capability of resource or nutrient utilization could synergize between fermentation strains and substrates in influencing nutritional components of fermented foods. The present study emphasizes the importance of the synergy between fermentation strains and substrates in influencing nutritional components of fermented foods. By uncovering novel aspects of probiotics metabolic pathways, particularly BCHA production, this study could inform the selection of fermentation strains and support the targeted production of BCHAs.

## Figures and Tables

**Table 1:** Alterations in metabolite levels within individual metabolic pathways, as indicated by the pathway map in Figure 5. Pathway analysis of Metaboanalyst 6.0 “https://www.metaboanalyst.ca/ (accessed on 18 September 2024)” was used to evaluate the statistical significance of the pathway alteration by reaction (* *p* < 0.05, ** *p* < 0.01, *** *p* < 0.001).

Strain	Metabolism	Pyruvate Reaction	BCAA Reaction		
			Valine	Leucine	Isoleucine
*L. fermentum* EFEL 6804	Glycine, serine, and threonine metabolism	-	Increased *	Increased **	Increased *
	Pyruvate metabolism	Increased ***	Increased *	Increased *	Increased *
	Butanoate metabolism	Increased **	Increased	Increased *	Increased
	Galactose metabolism	-	Increased **	Increased **	-
*L. salivarius* KGMB 2057	Glycine, serine, and threonine metabolism	-	-	-	-
	Pyruvate metabolism	Increased	-	-	-
	Butanoate metabolism	Increased ***	-	-	-
	Galactose metabolism	Increased	-	-	-
*L. sakei* subsp. *sakei* KACC 12414	Glycine, serine, and threonine metabolism	Increased **	Increased *	Increased **	Increased *
	Pyruvate metabolism	Increased **	Decreased	Decreased	Decreased
	Butanoate metabolism	Increased *	Decreased	Decreased	-
	Galactose metabolism	-	Decreased	Decreased	Decreased *

**Table 2:** List of microbial strains used in the present study (an asterisk (*) denotes strains utilized in both reaction mixture experiments and MRS cultivation experiments).

No.	Strain	Species	No.	Strain	Species
1	KCCM ^a^ 35469	*Limosilactobacillus fermentum*	20	KGMB 2057 *	*Ligilactobacillus salivarius*
2	KCTC ^b^ 3112	*Limosilactobacillus fermentum*	21	KGMB 2058	*Ligilactobacillus salivarius*
3	KCTC 5049	*Limosilactobacillus fermentum*	22	KGMB 5648	*Ligilactobacillus salivarius*
4	KCTC 5468	*Limosilactobacillus fermentum*	23	KGMB 6508	*Ligilactobacillus salivarius*
5	EFEL 6804 *	*Limosilactobacillus fermentum*	24	KACC ^d^ 12414 *	*Latilactobacillus sakei* subsp. *sakei*
6	C1	*Limosilactobacillus fermentum*	25	KACC 12421	*Latilactobacillus sakei* subsp. *sakei*
7	C2	*Limosilactobacillus fermentum*	26	KCTC 3598	*Latilactobacillus sakei* subsp. *sakei*
8	C5	*Limosilactobacillus fermentum*	27	CCUG ^e^ 43454	*Latilactobacillus sakei* subsp. *sakei*
9	C6	*Limosilactobacillus fermentum*	28	CCUG 48119	*Latilactobacillus sakei* subsp. *sakei*
10	C7	*Limosilactobacillus fermentum*	29	CCUG 49612	*Latilactobacillus sakei* subsp. *sakei*
11	C8	*Limosilactobacillus fermentum*	30	CCUG 54388	*Latilactobacillus sakei* subsp. *sakei*
12	C9	*Limosilactobacillus fermentum*	31	CCUG 60651	*Latilactobacillus sakei* subsp. *sakei*
13	C10	*Limosilactobacillus fermentum*	32	CCUG 30939	*Latilactobacillus sakei* subsp. *carnosus*
14	C11	*Limosilactobacillus fermentum*	33	CCUG 32077	*Latilactobacillus sakei* subsp. *carnosus*
15	C12	*Limosilactobacillus fermentum*	34	CCUG 34545	*Latilactobacillus sakei* subsp. *carnosus*
16	KCTC 3156	*Ligilactobacillus salivarius*	35	CCUG 42687	*Latilactobacillus sakei* subsp. *carnosus*
17	KCTC 3157	*Ligilactobacillus salivarius*	36	CCUG 46871	*Latilactobacillus sakei* subsp. *carnosus*
18	KCTC 5922	*Ligilactobacillus salivarius*	37	CCUG 48611	*Latilactobacillus sakei* subsp. *carnosus*
19	KGMB ^c^ 2053	*Ligilactobacillus salivarius*	38	CCUG 8045	*Latilactobacillus sakei* subsp. *carnosus*

^a^ KCCM, Korean Culture Center of Microorganisms. ^b^ KCTC, Korean Collection for Type Cultures. ^c^ KGMB, Korean Gut Microbiome Bank. ^d^ KACC, Korean Agricultural Culture Collection. ^e^ CCUG, Culture Collection University of Gothenburg.

## Data Availability

Additional supporting data related to this study are available from the corresponding author upon reasonable request. Source data for metabolite profiling in Supplementary Tables S1 and S2.

## Supplementary Materials

The following supporting information can be downloaded at: https://www.mdpi.com/article/10.3390/ijms251810112/s1.
