# Supplementary material for: Pathway and Production Differences in Branched-Chain Hydroxy Acids as Bioactive Metabolites in Limosilactobacillus fermentum, Ligilactobacillus salivarius, and Latilactobacillus sakei
Source: Int J Mol Sci. 2024 Sep 20;25(18):10112. doi: 10.3390/ijms251810112 (PMC11432647; doi:10.3390/ijms251810112)
Supplement: Supplementary file 1 [file ijms-25-10112-s001.zip › ijms-3203961-supplementary.pdf]

# *Supplementary Materials*

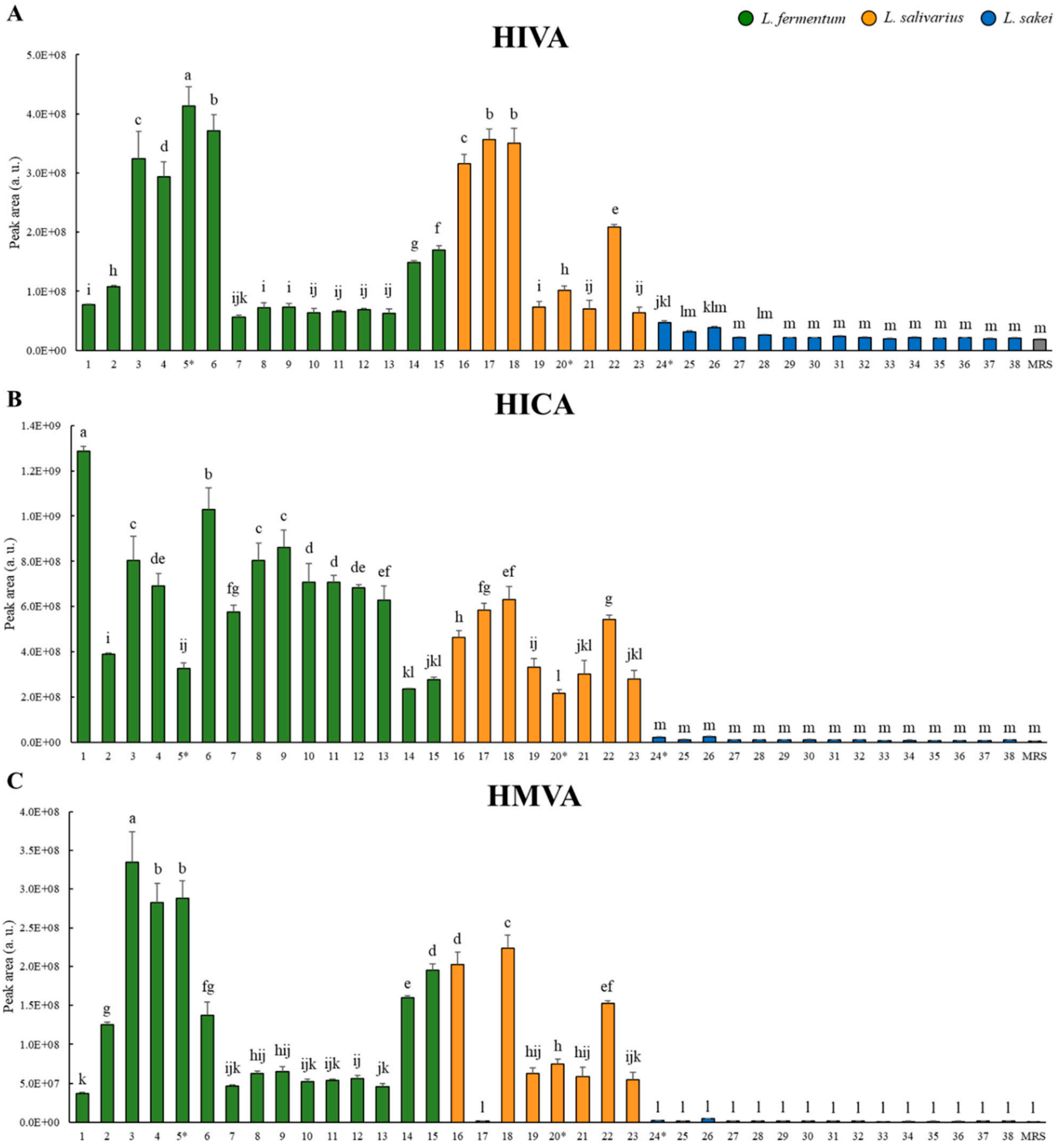


**Supplementary Figure 1.** BCHA production in MRS cultivation experiments with 38 strains of Lactobacillaceae are presented. The strain numbers correspond to Table 1 and non-fermented MRS sample data was presented. (A) Peak area of 2-hydroxy isovaleric acid (HIVA) (B) Peak area of 2-hydroxy isocaproic acid (HICA) for each strain. (C) Peak area of 2-hydroxy-3-methyl valeric acid (HMVA) for each strain. Asterisks indicate the strains utilized in reaction mixture experiment. Different letters in the bar graph indicate a significant difference determined by ANOVA followed by Duncan's multiple-range test ($p < 0.05$).

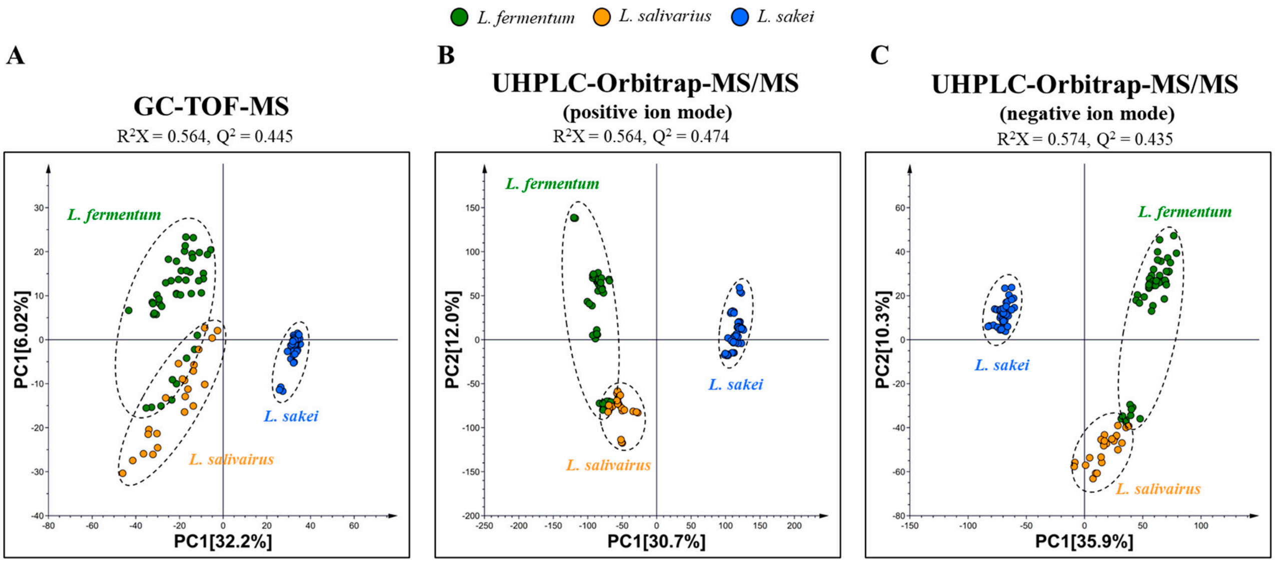


**Supplementary Figure 2.** Principal component analysis of the extracellular metabolome among 38 strains of Lactobacillaceae. Dataset (A) utilizes GC-TOF-MS analysis, while datasets (B) and (C) utilize UHPLC-Orbitrap-MS/MS in positive and negative ion modes, respectively. All samples underwent analytical instrument analysis with three repetitions.

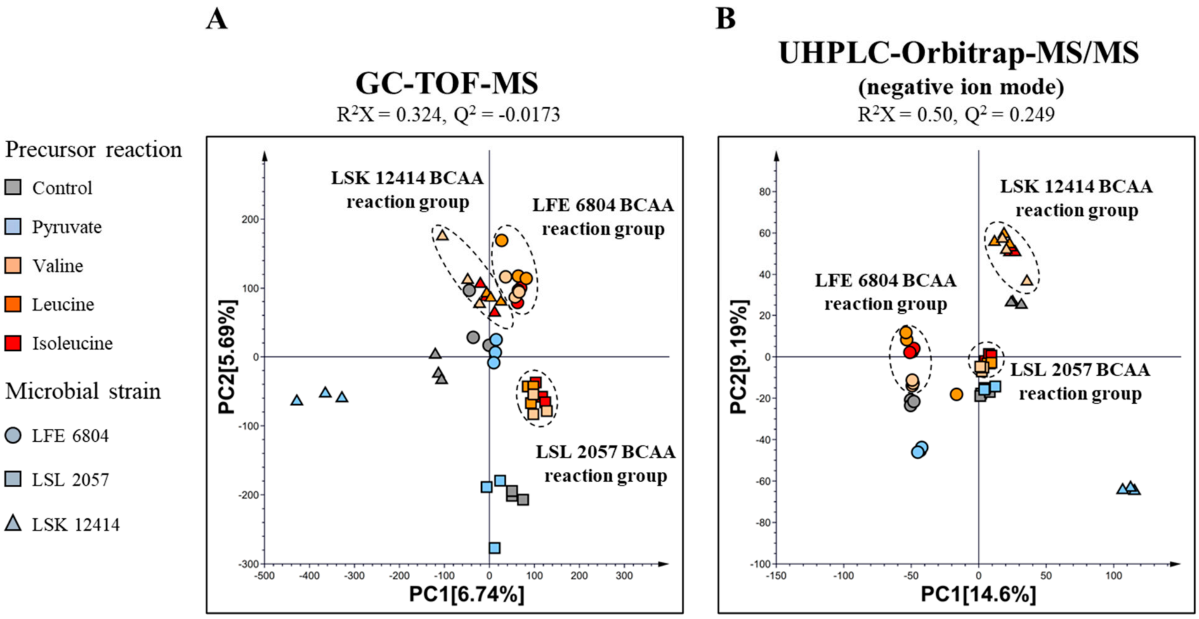


**Supplementary Figure 3.** Principal component analysis of the reaction mixture. Dataset (A) utilizes GC-TOF-MS analysis, while datasets (B) utilize UHPLC-Orbitrap-MS/MS in negative ion mode. LFE 6804, *Limosilactobacillus fermentum* EFEL 6804; LSL 2057, *Ligilactobacillus salivarius* KGMB 2057; LSK 12414, *Latilactobacillus sakei subsp. sakei* KACC 12414

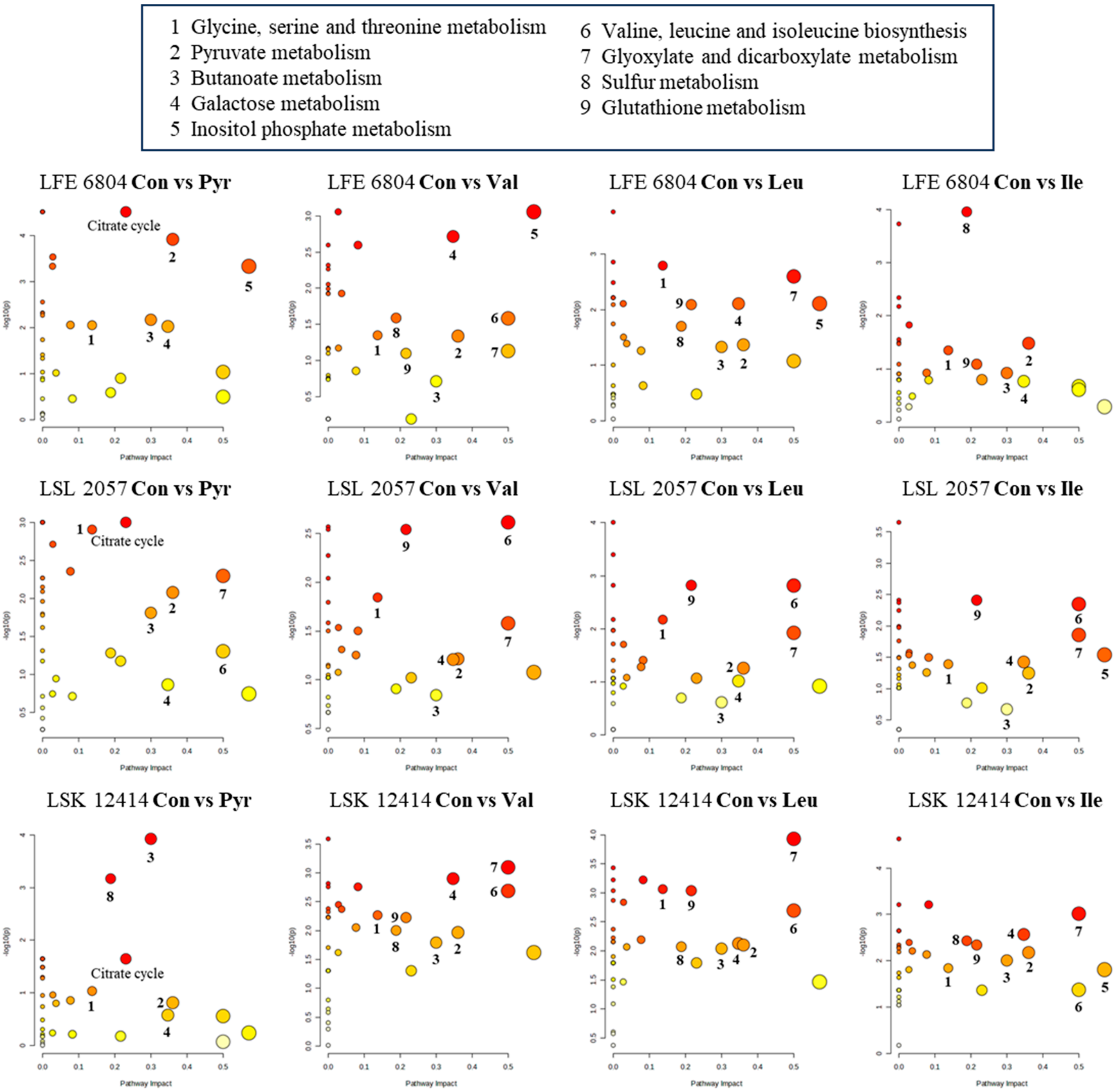


**Supplementary Figure 4.** Enrichment diagram of reaction mixtures. LFE 6804, *Limosilactobacillus fermentum* EFEL 6804; LSL 2057, *Ligilactobacillus salivarius* KGMB 2057; LSK 12414, *Latilactobacillus sakei subsp. sakei* KACC 12414

**Supplementary Table 1.** List of discriminant metabolites derived from GC-TOF-MS datasets of Lactobacillaceae three species reaction mixture experiments.

| No. | Tentative Identification | GC-TOF-MS | | | | | | | |
|---|---|---|---|---|---|---|---|---|---|
| | | One way ANOVA p-value of each strains | | | Ret (min) | Unique mass(m/z) | MS fragment pattern(m/z) | TMS | ID |
| | | LFE 6804 | LSL 2057 | LSK 12414 | | | | | |
| *Organic acids* | | | | | | | | | |
| 1 | Pyruvate | 0.000 | 0.058 | 0.000 | 5.22 | 174 | 73, 174, 74, 59, 89, 115, 72, 75, 99, 58 | 1 | Lib/STD |
| 2 | Lactate | 0.001 | 0.130 | 0.000 | 5.33 | 117 | 73, 147, 117, 73, 66, 191, 148, 74, 75, 190 | 2 | Lib/MS |
| 3 | Acetate | 0.001 | 0.118 | 0.000 | 5.50 | 177 | 73, 147, 66, 59, 148, 177, 205, 74, 133, 131 | 2 | Lib/MS |
| 4 | Succinate | 0.000 | 0.462 | 0.003 | 7.93 | 149 | 147, 75, 148, 149, 55, 56, 57, 72, 61, 172 | 2 | Lib/STD |
| 5 | Glycerate | 0.000 | 0.148 | 0.000 | 8.13 | 189 | 73, 147, 189, 103, 133, 292, 102, 74, 75, 117 | 3 | Lib/STD |
| 6 | 2-Hydroxy glutarate | 0.000 | 0.122 | 0.000 | 10.21 | 129 | 73, 129, 147, 75, 247, 157, 313, 85, 133, 203 | 3 | Lib/MS |
| 7 | Citrate | 0.000 | 0.244 | 0.000 | 12.09 | 273 | 73, 147, 273, 75, 74, 274, 149, 148, 347, 375 | 4 | Lib/STD |
| 8 | 4-Hydroxyphenyl lacitic acid | 0.004 | 0.125 | 0.000 | 12.63 | 179 | 179, 73, 180, 308, 75, 74, 181, 133, 281, 149 | 3 | Lib/MS |
| 9 | Gluconate | 0.002 | 0.754 | 0.000 | 13.18 | 147 | 73, 147, 217, 333, 103, 205, 292, 74, 75, 319 | 6 | Lib/STD |
| *Alchols* | | | | | | | | | |
| 10 | 2,3-Butanediol | 0.000 | 0.035 | 0.000 | 5.10 | 117 | 117, 73, 147, 118, 75, 74, 66, 148, 59, 133 | 2 | Lib/MS |
| 11 | Myo-Inositol | 0.001 | 0.226 | 0.000 | 13.95 | 217 | 73, 217, 147, 305, 191, 318, 75, 204, 129, 74 | 6 | Lib/STD |
| *Amino acids & amino acid derivatives* | | | | | | | | | |
| 12 | Alanine | 0.001 | 0.030 | 0.000 | 5.81 | 116 | 116, 73, 117, 74, 59, 118, 100, 190, 218, 94 | 2 | Lib/STD |
| 13 | Valine | 0.000 | 0.000 | 0.000 | 7.01 | 144 | 144, 218, 145, 72, 100, 146, 219, 128, 156, 220 | 2 | Lib/STD |
| 14 | Leucine | 0.000 | 0.048 | 0.000 | 7.56 | 158 | 158, 159, 102, 100, 160, 232, 142, 260, 186, 108 | 2 | Lib/STD |
| 15 | Isoleucine | 0.000 | 0.001 | 0.000 | 7.75 | 158 | 158, 73, 218, 159, 147, 74, 86, 100, 59, 160 | 2 | Lib/STD |
| 16 | Glycine | 0.000 | 0.002 | 0.000 | 7.92 | 174 | 174, 73, 86, 175, 248, 100, 59, 176, 133, 74 | 3 | Lib/STD |
| 17 | Butyrylglycine | 0.000 | 0.015 | 0.002 | 10.17 | 158 | 73, 158, 202, 75, 100, 74, 147, 304, 159, 203 | 1 | Lib/STD |
| *Carbohydrates* | | | | | | | | | |
| 18 | Carbohydrate 1 | 0.000 | 0.249 | 0.000 | 11.09 | 103 | 73, 103, 147, 217, 307, 75, 189, 74, 117, 133 | 4 | Lib/MS |
| 19 | Carbohydrate 2 | 0.401 | 0.309 | 0.000 | 11.75 | 147 | 73, 147, 103, 129, 292, 75, 217, 74, 117, 205 | 5 | Lib/MS |
| 20 | Arabino-hexonic acid | 0.000 | 0.573 | 0.000 | 11.79 | 129 | 73, 129, 147, 75, 155, 103, 205, 246, 133, 117 | 3 | Lib/MS |
| 21 | Carbohydrate 3 | 0.084 | 0.071 | 0.000 | 12.24 | 217 | 73, 217, 147, 260, 133, 191, 159, 247, 305, 129 | 6 | Lib/MS |
| 22 | Fructose | 0.009 | 0.180 | 0.000 | 12.51 | 217 | 73, 103, 217, 147, 307, 75, 74, 133, 117, 104 | 5 | Lib/STD |
| 23 | Galactose | 0.000 | 0.111 | 0.000 | 12.69 | 205 | 73, 205, 147, 319, 160, 103, 217, 75, 74, 320 | 5 | Lib/STD |
| 24 | Sucrose | 0.098 | 0.132 | 0.000 | 17.01 | 361 | 73, 361, 217, 147, 103, 129, 362, 169, 271, 74 | 8 | Lib/STD |
| *Fatty acids & lipids* | | | | | | | | | |

| | | | | | | | | | |
|---|---|---|---|---|---|---|---|---|---|
| 25 | Hexanoic acid | 0.006 | 0.196 | 0.000 | 5.43 | 173 | 75, 73, 173, 117, 131, 74, 69, 55, 132, 76 | 1 | Lib/MS |
| 26 | 2-Hydroxy isovaleric acid | 0.000 | 0.015 | 0.000 | 6.48 | 145 | 73, 145, 147, 148, 219, 146, 133, 149, 73, 247 | 2 | Lib/STD |
| 27 | 2-Hydroxy isocaproic acid | 0.000 | 0.001 | 0.000 | 7.19 | 159 | 73, 159, 103, 147, 75, 69, 160, 74, 133, 59 | 2 | Lib/STD |
| 28 | 2-Hydroxy-3-methylvaleric acid | 0.000 | 0.000 | 0.000 | 7.24 | 159 | 73, 159, 147, 75, 69, 103, 160, 74, 117, 148 | 2 | Lib/MS |
| 29 | 2-Hydroxy hexanoic acid | 0.000 | 0.000 | 0.000 | 7.27 | 159 | 159, 147, 69, 160, 133, 148, 149, 233, 161, 131 | 2 | Lib/MS |
| 30 | Nonanoic acid | 0.004 | 0.220 | 0.000 | 8.35 | 215 | 75, 73, 117, 215, 55, 132, 129, 57, 131, 74 | 1 | Lib/STD |
| 31 | Myristic acid | 0.033 | 0.244 | 0.000 | 12.19 | 117 | 73, 75, 117, 132, 57, 55, 285, 145, 71, 69 | 1 | Lib/STD |
| 32 | Palmitic Acid | 0.012 | 0.212 | 0.000 | 13.47 | 117 | 73, 117, 75, 132, 129, 55, 313, 145, 57, 131 | 1 | Lib/STD |
| 33 | Monopalmitin | 0.003 | 0.234 | 0.000 | 16.36 | 129 | 129, 73, 218, 147, 103, 57, 75, 55, 203, 131 | 2 | Lib/MS |
| 34 | Monostearin | 0.002 | 0.257 | 0.000 | 17.31 | 129 | 129, 73, 218, 147, 103, 75, 57, 55, 131, 203 | 2 | Lib/MS |
| *Pyrimidine* | | | | | | | | | |
| 35 | Uracil | 0.000 | 0.000 | 0.000 | 9.09 | 179 | 75, 179, 136, 78, 51, 76, 105, 180, 77, 61 | 1 | Lib/MS |
| *Unknowns* | | | | | | | | | |
| 36 | N.I 1 | 0.000 | 0.000 | 0.000 | 8.33 | 140 | 140, 89, 77, 59, 61, 58, 69, 60, 134, 200 | 0 | Lib/MS |
| 37 | N.I 2 (Phenolic compound) | 0.000 | 0.195 | 0.000 | 8.43 | 188 | 188, 73, 100, 147, 59, 189, 262, 114, 133, 190 | 0 | Lib/MS |

[1] Discriminant metabolites were determined by ANOVA ($p < 0.05$) at least only one strain satisfies the requirement. Discriminant metabolites were determined by ANOVA ($p < 0.05$) when at least one strain satisfies the requirement.
[2] LFE 6804, *Limosilactobacillus fermentum* EFEL 6804.
[3] LSL 2057, *Ligilactobacillus salivarius* KGMB 2057.
[4] LSK 12414, *Latilactobacillus sakei subsp. sakei* KACC 12414.

**Supplementary Table 2.**List of discriminant metabolites derived from UHPLC-Orbitrap-MS/MS datasets of Lactobacillaceae three species reaction mixture experiments.

| No. | Tentative Identification[a] | UHPLC-Orbitrap-MS/MS | | | | | | | | | | |
|---|---|---|---|---|---|---|---|---|---|---|---|---|
| | | One way ANOVA p-value of each strain | | | RT (min) | [M-H]⁻ | [M+H]⁺ | M.W. | Molecular Formula | Error (ppm) | MS[2] fragment pattern (*m/z*) | Identification Reference |
| | | LFE 6804 | LSL 2057 | LSK 12414 | | | | | | | | |
| *Organic acids* | | | | | | | | | | | | |
| 1 | Aminoadipic acid | 0.001 | 0.000 | 0.003 | 0.81 | 160.0615 | - | 161 | C6H11NO4 | -0.37 | - | Patricia et al. 2021 |
| 2 | Glutarate | 0.000 | 0.000 | 0.001 | 0.88 | 131.0352 | - | 132 | C5H8O4 | 1.68 | (-)113, 87 | Zhng et al. 2010 |
| 3 | Phenyllactic acid | 0.001 | 0.000 | 0.002 | 5.13 | 165.0558 | - | 166 | C9H10O3 | 0.23 | (-)147, 119, 73 | Kim et al. 2023 |
| 4 | Indolelactic acid | 0.002 | 0.000 | 0.029 | 5.65 | 204.0665 | - | 205 | C11H11NO3 | -0.66 | (-)186, 158, 116 | Kim et al. 2023 |
| *Amino acids & amino acid derivatives* | | | | | | | | | | | | |
| 5 | Phenylalanine | 0.000 | 0.000 | 0.000 | 0.82 | - | 166.0860 | 165 | C9H11NO2 | -1.45 | (+)120, 85 | Wang et al. 2018 |
| 6 | N-acetyl-aspartic acid | 0.004 | 0.000 | 0.006 | 0.82 | 174.0408 | - | 175 | C6H9NO5 | -0.14 | (-)156, 130, 88 | Kim et al. 2023 |
| 7 | Tryptophan | 0.000 | 0.060 | 0.000 | 0.92 | - | 205.0966 | 204 | C11H12N2O2 | -1.19 | (+)188, 146 | Wang et al. 2018 |
| 8 | Pyroglutamyl valine | 0.739 | 0.036 | 0.034 | 0.94 | 227.1036 | - | 228 | C10H16N2O4 | -0.47 | (-)183, 127, 82 | Kim et al. 2023 |
| 9 | Lactoyl-phenylalanine | 0.600 | 0.002 | 0.275 | 5.61 | 236.0927 | - | 237 | C12H15O4N | -0.37 | - | Kim et al. 2023 |
| 10 | Nonanoylglycine | 0.000 | 0.000 | 0.001 | 6.52 | 214.1449 | 216.1593 | 215 | C11H21O3N | 0.07 | 197, 195, 178 | Kim et al. 2023 |
| *Fatty acids* | | | | | | | | | | | | |
| 11 | 2-Hydroxy-4-(methylthio)butanoic acid | 0.000 | 0.000 | 0.708 | 1.11 | 149.0280 | - | 150 | C5H10O3S | 1.42 | - | Kim et al. 2023 |
| *Purines & pyrimidines* | | | | | | | | | | | | |
| 12 | Xanthine | 0.031 | 0.000 | 0.001 | 0.82 | 151.0261 | - | 152 | C5H4N4O2 | 0.13 | (-)108, 42 | Kim et al. 2023 |
| *Unknowns* | | | | | | | | | | | | |
| 13 | N. I 5 | 0.000 | 0.037 | 0.001 | 0.67 | 175.0249 (M+FA-H) | - | 130 | C5H6O4 | 0.35 | (-)113, 87, 69 | |
| 14 | N. I 3 | 0.089 | 0.433 | 0.005 | 0.90 | 161.0458 | - | 162 | C6H10O5 | 1.33 | (-)117, 89, 71 | |
| 15 | N. I 4 | 0.000 | 0.000 | 0.000 | 10.82 | - | 414.3207 | 413 | C23H43NO5 | -1.74 | (+)271, 165, 133, 89 | |

[1] Discriminant metabolites were determined by ANOVA ($p < 0.05$) when at least one strain satisfies the requirement.
[2] LFE 6804, *Limosilactobacillus fermentum* EFEL 6804.
[3] LSL 2057, *Ligilactobacillus salivarius* KGMB 2057.
[4] LSK 12414, *Latilactobacillus sakei* subsp. *sakei* KACC 12414.
